# Mice Deficient in the IL-1β Activation Genes *Prtn3*, *Elane*, and *Casp1* Are Protected Against the Development of Obesity-Induced NAFLD

**DOI:** 10.1007/s10753-020-01190-4

**Published:** 2020-01-30

**Authors:** Andreea-Manuela Mirea, Rinke Stienstra, Thirumala-Devi Kanneganti, Cees J. Tack, Triantafyllos Chavakis, Erik J.M. Toonen, Leo A.B. Joosten

**Affiliations:** 1 Department of Internal Medicine, Radboud Institute for Molecular Life Sciences (RIMLS), Radboud University Medical Center, Geert Grooteplein Zuid 8, 6525 GA Nijmegen, The Netherlands; 2 Department of Medical Genetics, Iuliu Hatieganu University of Medicine and Pharmacy, Pasteur Street, Number 6, 400349 Cluj-Napoca, Romania; 3 Nutrition, Metabolism and Genomics Group, Division of Human Nutrition, Wageningen University, Droevendaalsesteeg 4, 6708 PB Wageningen, The Netherlands; 4 Department of Immunology, St Jude Children’s Research Hospital, Memphis, TN 38105 USA; 5 Institute for Clinical Chemistry and Laboratory Medicine, University Clinic Carl-Gustav-Carus, Technische Universität Dresden, Fetscherstraße 74, 01307 Dresden, Germany; 6 Paul Langerhans Institute Dresden, Helmholtz Zentrum München, University Hospital and Faculty of Medicine Carl Gustav Carus, TU Dresden, Tatzberg 47, 01307 Dresden, Germany; 7 German Center for Diabetes Research (DZD e.V.), Neuherberg, Germany; 8 R&D Department, Hycult Biotechnology, Frontstraat 2A, 5405 PB Uden, The Netherlands

**Keywords:** obesity, inflammation, neutrophil serine proteases, IL-1 beta

## Abstract

Non-alcoholic fatty liver disease (NAFLD) is the most common cause of chronic liver disease. Inflammatory pathways contribute to disease pathogenesis; however, regulation of the underlying mechanism is not completely understood. IL-1β, a pro-inflammatory cytokine, participates in the development and progression of NAFLD. To become bioactive, IL-1β requires enzymatic processing. Mechanisms that activate IL-1β include the classical NLRP3 inflammasome-caspase-1 and the neutrophil serine proteases, neutrophil elastase, and proteinase-3. Several studies have shown that both caspase-1 and the neutrophil serine proteases are important for NAFLD development. However, it is unknown whether these pathways interact and if they have a synergistic effect in promoting NAFLD. In the present study, we developed a novel and unique mouse model by intercrossing caspase-1/11 knockout mice with neutrophil elastase/proteinase-3 double knockout mice. Subsequently, these mice were examined regarding the development of high-fat diet–induced NAFLD. Our results show that mice deficient in caspase-1, neutrophil elastase, and proteinase-3 were protected from developing diet-induced weigh gain, liver steatosis, and adipose tissue inflammation when compared with controls. We conclude that pathways that process pro-IL-1β to bioactive IL-1β play an important role in promoting the development of NAFLD and obesity-induced inflammation. Targeting these pathways could have a therapeutic potential in patients with NAFLD.

**Electronic supplementary material:**

The online version of this article (10.1007/s10753-020-01190-4) contains supplementary material, which is available to authorized users.

## INTRODUCTION

**Fig. 1:**
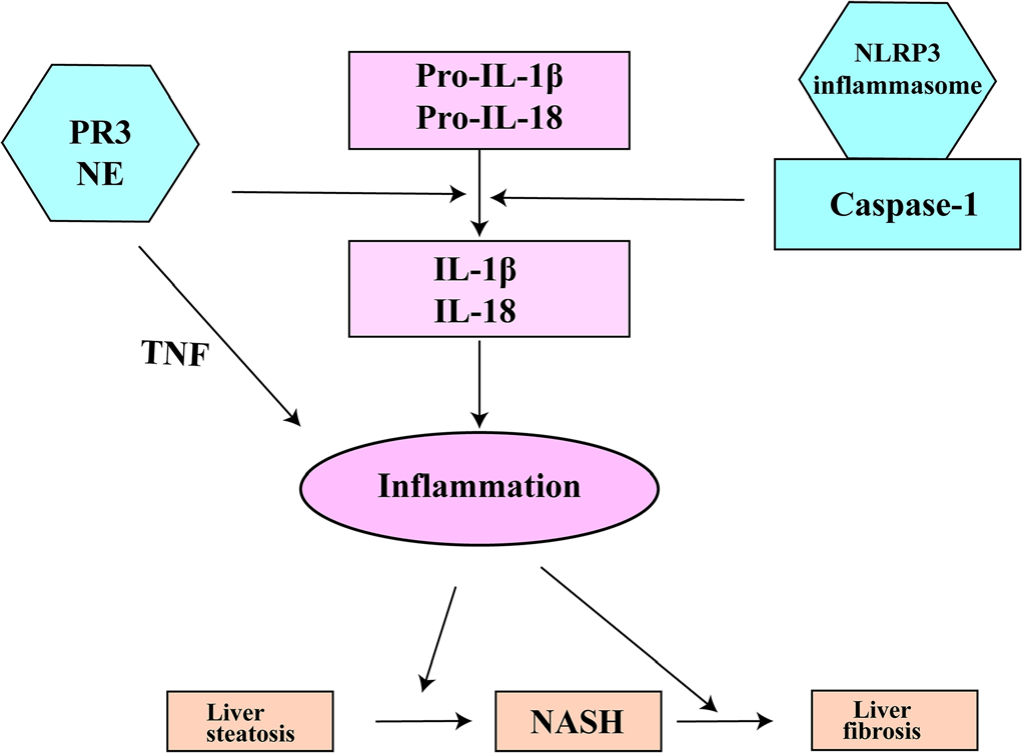
The role of IL-1β activation pathways in NAFLD. Pro-inflammatory cytokines contribute to NAFLD development from liver steatosis to NASH and fibrosis by activating sterile inflammation in the liver. Some of these cytokines such as IL-1β and IL-18 are secreted as inactive and need proteolytic cleavage in order to become active. Two mechanisms responsible for IL-1 cytokine activation are represented by the NLRP3 inflammasome-caspase-1 protein complex and the neutrophil serine proteases PR3 and NE. Additionally to IL-1β and IL-18, PR3 and NE are able to activate membrane-bound TNF. Activating pro-inflammatory NLRP3 inflammasome-caspase-1 complex and the neutrophil serine proteases also contributes to NAFLD development and progression. IL-1β, interleukin-1β; IL-18, interleukin-18; NASH, non-alcoholic steatohepatitis; NE, neutrophil elastase; PR3, proteinase-3; Pro-IL-1β, pro-interleukin-1β; Pro-IL-18, pro-interleukin-18; TNF, tumor necrosis factor.

Non-alcoholic fatty liver disease (NAFLD) is the most common cause of chronic liver disease worldwide [1]. It is strongly associated with obesity and insulin resistance [2]. NAFLD ranges from mild liver steatosis to severe liver steatosis accompanied by hepatic inflammation, in which case the term non-alcoholic steatohepatitis (NASH) is used. NASH can further progress into liver fibrosis, cirrhosis, and even hepatocellular carcinoma [3]. Although the disease is frequent, the underlying mechanisms responsible for its development and progression are not fully understood. During the last few years, it became apparent that inflammation is an important factor in the pathogenesis of NAFLD. The pro-inflammatory cytokine IL-1β has a key role in the development and progression of NAFLD being involved in all the stages ranging from liver steatosis to NASH and fibrosis [4]. IL-1β is produced as an inactive protein and needs proteolytic cleavage in order to become bioactive. Several mechanisms are able to activate IL-1β in NAFLD. Most studied for its role in NAFLD and another metabolic disturbance is the classical NLRP3 inflammasome activation pathway [5–8]. Upon stimulation, the NLRP3 protein complex assembles and cleaves pro-caspase-1 to active caspase-1. In turn, caspase-1 activates the pro-inflammatory cytokines IL-1β and IL-18 leading to induction and/or prolongation of inflammation [9] (Fig. 1). Several studies have shown that the NLRP3 inflammasome and caspase-1 are directly involved in NAFLD by promoting the development of inflammation and fibrosis and also indirectly by promoting associated metabolic disturbances such as adipose tissue inflammation and insulin resistance [6, 7, 10–12]. However, next to the NLRP3 inflammasome-caspase-1 pathway, also neutrophil serine proteases (NSPs), such as proteinase 3 (PR3) and neutrophil elastase (NE), are able to process cytokines to their bioactive forms. These neutrophil serine proteases are stored in the azurophilic granules of neutrophils. Upon neutrophil activation, these proteases are released and activate several cytokines, such as IL-1β, IL-18, and TNF [13], either in the neutrophil cytosol or in the extracellular compartment [14] (Fig. 1). When extracellular, NSPs are inhibited by alpha-1 antitrypsin (AAT), an acute phase protein mainly synthesized in the liver [15], thereby attenuating the inflammatory response. Recently, several studies have shown that these NSPs are involved in the development of metabolic diseases such as T2DM and NAFLD in an inflammasome-independent manner. For example, Mansuy-Aubert and collaborators have shown that NE knockout mice are protected from developing obesity-induced liver steatosis and insulin resistance [16]. Moreover, they have shown that there is an imbalance between NE serum activity and AAT serum concentrations in obese individuals [16]. Consistent with these results, Zang and collaborators have shown an imbalance between NE and AAT serum concentrations in individuals with biopsy-diagnosed NAFLD when compared with healthy individuals. They calculated rather good sensitivity and specificity scores for the NE/AAT ratio in order to predict the advanced stages of the disease [17]. Our group has recently shown that, additionally to NE, also PR3 plays an important role in the development of obesity-induced NAFLD and insulin resistance in a mouse model for obesity-induced NAFLD [18]. Furthermore, we have shown that administering human AAT protects against disease development in a mouse model of obesity-induced NAFLD [18].

These studies clearly indicate that both inflammasome-dependent and independent pathways are involved in the development of metabolic conditions. The aim of this study was to investigate the synergistic effect of NSPs and the inflammasome/caspase-1 on the development of NAFLD. To do so, we developed a unique knockout mouse model deficient in caspase-1 (*Casp1*), PR3 (*Prtn3*), and NE (*Elane*), in which the development of diet-induced NAFLD was assessed.

## MATERIAL AND METHODS

### Animals

Caspase-1 (*Casp1*) knockout mice, which also miss caspase-11 (*Casp11*) [19] and NE/PR3 (*Prtn3/Elane*) knockout mice, were developed as previously described [20, 21] and intercrossed to obtain a quadruple knockout mice (Casp1/Casp11/NE/PR3). Genotyping of the mice was performed as previously described [19, 20]. Age-matched wild-type (WT) C57BL/6J mice were used as controls.

Animals were single-caged in a pathogen-free facility with a 12:12-h light-dark cycle and water and food *ad libitum*. All mice were 10- to 12-week-old males at the start of diet intervention. All interventions were approved by the Ethics Committee for Animal Experiments at Wageningen University. All experiments were conducted in accordance with the EU Directive 2010/63 for animal experiments.

### Diet Intervention

Both male Casp1/Casp11/NE/PR3 knockout and male WT control mice were divided in groups of 12 animals and given a low-fat diet (LFD) or a high-fat diet (HFD) with 10%, or 45% of the energy derived from fat, respectively (D12450B, D12451; Research Diets), for 16 weeks. Body weight and food intake were measured weekly.

Insulin tolerance test (ITT) and oral glucose tolerance test (oGTT) were performed to assess glycemic control. Mice were fasted 5 h before intraperitoneal administration of insulin (0.75 U/kg) or glucose administration (2 g/kg) by oral gavage. Blood samples were obtained by tail-cut and glucose concentration was measured at baseline and after 15, 30, 45, 60, 90, and 120 min using an Accu-Chek glucose meter (Roche Diagnostics, Almere, The Netherlands).

### Plasma Measurements

At the end of the study, blood was collected in heparin-coated tubes and centrifuged to collect plasma. Triglycerides (TG) and total cholesterol (TC) were determined using a liquicolor kit from Instruchemie (Delfzijl, The Netherlands). Free fatty acids (FFAs) were measured using the NEFA-C kit from Wako Diagnostics (Instruchemie, Delfzijl, The Netherlands). Glucose was measured enzymatically following manufacturer’s protocols (Liquicolor, Human GmbH, Wiesbaden, Germany). Adiponectin and leptin were measured by ELISA using manufacturer’s protocol (R&D Systems, Minneapolis, USA). All samples were measured in a single replicate. For each run, in-house controls were included in order to test for reproducibility and batch-to-batch variation.

### Liver and Gonadal Adipose Tissue (gWAT) Histology

Liver and gWAT were isolated, formalin-fixed, and paraffin-embedded before staining. Liver sections of 5 μm were cut and stained with hematoxylin and eosin. Liver steatosis was determined by assessing the percentage of cells containing lipid droplets per field. Four fields were analyzed per sample. We noted with “0” the absence of liver steatosis. Steatosis degrees were defined as follows: first degree (noted with “1”)—between 5 and 33% of cells per field contained lipid droplets; second degree (noted with “2”)—between 33 and 66% of cells per field contained lipid droplets; third degree (noted with “3”)—more than 66% of cells per field contained lipid droplets [22]. Gonadal adipose tissue sections of 8 μm were cut and stained by DAB (3′3-diaminobenzidine) technique with an F4/80 antibody (Bio-Rad, Veenendaal, The Netherlands). Crown-like structures and adipocytes were counted in four images per sample.

### Liver Triglycerides Assessment

To measure liver triglycerides, we prepared 20% liver homogenates using a buffer with 0.25 M saccharose, 20 mM Tris-HCL, 1 mM dithiothreitol (DTT), and 1% Triton X-100 at a pH of 7.4. Triglycerides were measured using a liquicolor kit from Instruchemie (Delfzijl, The Netherlands). All samples were measured in a single replicate. For each run, in-house controls were included in order to test for reproducibility and batch-to-batch variation.

### RNA Isolation and Gene Expression Analysis

Total RNA was isolated from liver and gWAT using TRIzol reagent (Life Technologies Europe BV, Bleiswijk, The Netherlands) and quantified with a NanoDrop spectrophotometer (NanoDrop Technologies, Wilmington, USA). A total of 1 μg of RNA was reverse-transcribed to cDNA using an iScript mix (Bio-Rad Laboratories) and gene expression analysis was done by quantitative polymerase chain reaction (qPCR) using SYBR green–based quantification (Applied Biosystems, Foster City, CA, USA). Primers were developed with Primer3 (Primer3 Input (version 0.4.0) or selected from Harvard Primer Bank (https://pga.mgh.harvard.edu/primerbank/).The gene *36b4* was used as an endogenous control. Differences in expression were calculated using the 2^ΔΔCt^ method [23]. A list with our primers’ sequence is available in Supplementary Table 1. All samples were measured in duplicates.

### Statistical Analysis

Data are represented as mean ± SEM. Statistical analysis and graphs were performed using Graphpad Prism 5.03 (La Jolla, USA). Data were analyzed using, as appropriate, the Student *t* test or one-way ANOVA with Tukey *post hoc* test. To examine the effects of both diet and genotype in our murine model, we used a two-way ANOVA with Bonferroni *post hoc* test. A *p* value < 0.05 was considered significant.

## RESULTS

### Deficiency in IL-1β Activation Pathways Protects Against HFD-Induced Obesity

**Fig. 2:**
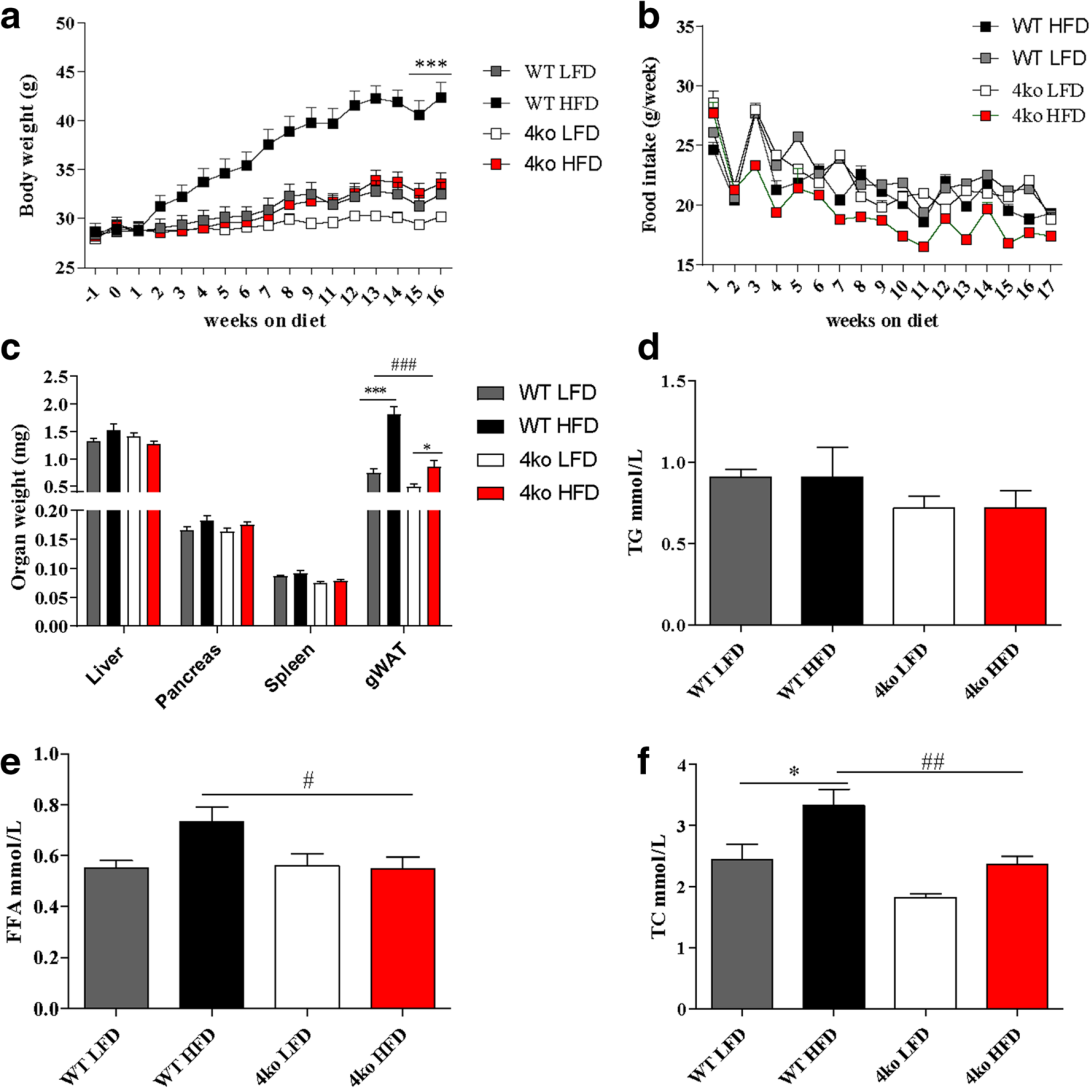
Phenotype at the end of the diet. WT, wild-type mice; 4ko, Casp1/Casp11/NE/PR3 knockout mice. **a** Gain in body weight during the HFD/LFD intervention. **b** Weekly food intake for the four groups of mice. **c** Liver, pancreas, spleen, and gWAT weight at the end of diet intervention. **d** Plasma concentrations of triglycerides (TG) at the end of diet intervention. **e** Plasma concentrations of free fatty acids at the end of diet intervention. **f** Plasma concentrations of total cholesterol (TC) at the end of diet intervention. All samples were measured in a single replicate. Data is represented as mean ± SEM. *p < 0.05, effect of the diet; ***p < 0.001, effect of the diet; #*p* < 0.05, effect of the genotype; ##*p* < 0.01, effect of the genotype; ###*p* < 0.001, effect of the genotype.

To explore the combined effect of NSPs and the inflammasome/caspase-1 pathway on NAFLD disease development, Casp1/Casp11/NE/PR3 knockout mice and WT control mice were fed a LFD or a HFD for 16 weeks. Before the start of the diet intervention, no differences in bodyweight were observed between the groups. As expected, bodyweight of the WT mice group increased significantly after HFD (Fig. 2a). In the Casp1/Casp11/NE/PR3 knockout mice, weight gain was significantly less after HFD when compared with the WT group (*p* < 0.0001) and was similar to the weight gain observed in the WT and the Casp1/Casp11/NE/PR3 mice fed a LFD (Fig. 2a). No differences in food intake were observed between Casp1/Casp11/NE/PR3 knockout mice and WT mice fed a HFD (Fig. 2b). Mice that received a HFD had a significant higher gWAT weight when compared with mice fed the LFD (1.81 ± 0.42 mg *versus* 0.74 ± 0.27 mg, *p* < 0.0001 for WT mice and 0.86 ± 0.39 mg *versus* 0.49 mg ± 0.19 mg, *p* < 0.05 for Casp1/Casp11/NE/PR3 knockout mice) (Fig. 2c). No differences in liver, spleen, and pancreas weight were observed between the four mice groups. Notably, WT mice fed a HFD had a higher gWAT weight than Casp1/Casp11/NE/PR3 knockout mice fed the same diet (1.81 ± 0.42 mg *versus* 0.86 ± 0.39 mg; *p* < 0.0001). These results indicate that Casp1/Casp11/NE/PR3 knockout mice are protected from diet-induced obesity.

### Casp1/Casp11/NE/PR3 Knockout Mice Have Lower Circulating Lipid Levels

Circulating lipid levels are increased in obesity and are associated with cardiovascular and NAFLD outcome [24]. Therefore, we measured plasma lipid levels in the different genotypes to assess dyslipidemia (Fig. 2d–f). No differences in plasma triglycerides levels were observed between WT mice and 4ko mice, regardless of the type of diet. Interestingly, Casp1/Casp11/NE/PR3 knockout mice fed a HFD had significantly lower circulating free fatty acid levels (0.54 ± 0.16 mmol/L *versus* 0.7 ± 0.17 mmol/L; *p* = 0.02) (Fig. 2e) and lower circulating cholesterol levels (2.36 ± 0.43 mmol/L *versus* 3.32 ± 0.84 mmol/L; *p* < 0.01) (Fig. 2f) when compared with WT mice fed the same diet. Hence, Casp1/Casp11/NE/PR3 knockout mice had lower circulating lipid levels when compared with the WT controls.

### Casp1/Casp11/NE/PR3 Knockout Mice Are Protected from Developing Obesity-Induced Liver Steatosis

**Fig. 3:**
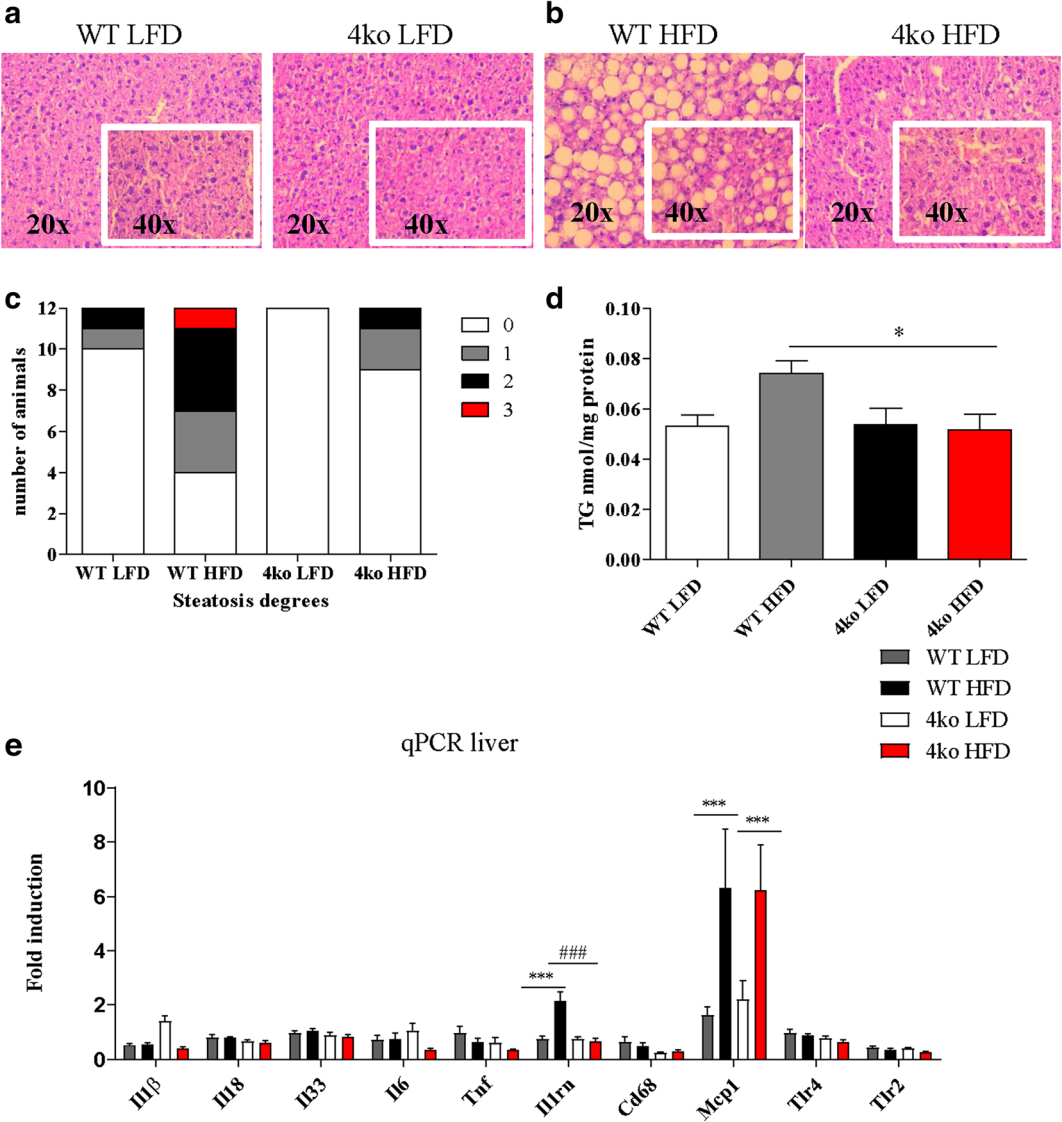
Liver status at the end of the diet. WT, wild-type mice; 4ko, Casp1/Casp11/NE/PR3 knockout mice. **a** Representative images of liver histology in the groups that received LFD at × 20 magnification, respectively × 40 magnification. **b** Representative image of liver histology in the groups that received HFD at × 20 magnification, respectively × 40 magnification. **c** Number of animals affected by different degrees of liver steatosis. **d** Triglyceride content of the liver in the four groups of mice. All samples were measured in a single replicate. **e** mRNA fold induction of several genes in the liver in the four groups of mice. All samples were measured in duplicates. Data is represented as mean ± SEM. *p < 0.05, effect of the diet; ***p < 0.001, effect of the diet; #*p* < 0.05, effect of the genotype; ###*p* < 0.001, effect of the genotype.

Liver steatosis was assessed in liver slides stained with hematoxylin and eosin (H&E). Non-quantified visual inspection of H&E-stained liver sections showed that lipid droplets were much more abundant in the livers of WT mice when compared with those of the knockout mice (Fig. 3a). In order to investigate the effect of diet intervention in the liver, we further quantified the degree of liver steatosis in the different mouse groups by assessing the percentage of cells containing lipid droplets per field in the stained liver slides. Nine of the Casp1/Casp11/NE/PR3 knockout mice fed a HFD (nine knockout mice *versus* four WT mice) did not develop liver steatosis (degree 0) (Fig. 3c). The remaining mice in this group also had less severe liver injury when compared with the WT HFD group (two Casp1/Casp11/NE/PR3 knockout with steatosis degree 1 and one knockout mouse with steatosis degree 2 *versus* three WT mice with steatosis degree 1, four WT mice with steatosis degree 2, and one WT mouse with steatosis degree 3) (Fig. 3c). Additionally, Casp1/Casp11/NE/PR3 knockout mice had significantly less triglyceride content in the liver when compared with WT controls (Fig. 3d). These results suggest that Casp1/Casp11/NE/PR3 knockout mice were protected from developing diet-induced liver steatosis.

Subsequently, we investigated mRNA expression profiles of several inflammatory markers in liver tissue. First, we assessed the mRNA levels of cytokines that are processed by both caspase-1 and/or NE and PR3, namely *Il1β*, *Il18*, and *Il33* [25] (Fig. 3e), but no differences between the mice groups were observed. Next, the mRNA levels for the pro-inflammatory cytokines *Tnf* and *Il6* and the anti-inflammatory marker *Il1rn* were measured (Fig. 3e). No difference was observed for *Tnf* and *Il6* between the mice groups. This suggests that the HFD did not induce pro-inflammatory cytokine transcription for these markers.

*Il1rn* (the gene that encodes for IL-1 receptor antagonist-IL1-Ra) mRNA levels were significantly higher in the WT mice that received a HFD when compared with those in the other groups (2.15-fold change *versus* 0.66-fold change, *p* < 0.001), thereby confirming that HFD induced a certain degree of hepatic inflammation. We further tested markers that are also increased during inflammation, including *Cd68*, *Mcp1* (monocyte chemoattractant protein-1), *Tlr2* (toll-like receptor 2), and *Tlr4* (toll-like receptor 4) (Fig. 3e). *Mcp1* mRNA levels were significantly increased in both knockout and WT mice that received a HFD when compared with the LFD groups, thereby suggesting induction of inflammation in the HFD groups. Altogether, no significant differences in mRNA expression levels were observed between WT and knockout for these inflammatory markers in liver tissue.

### Casp1/Casp11/NE/PR3 Knockout Mice Display Reduced Obesity-Induced Inflammation

**Fig. 4:**
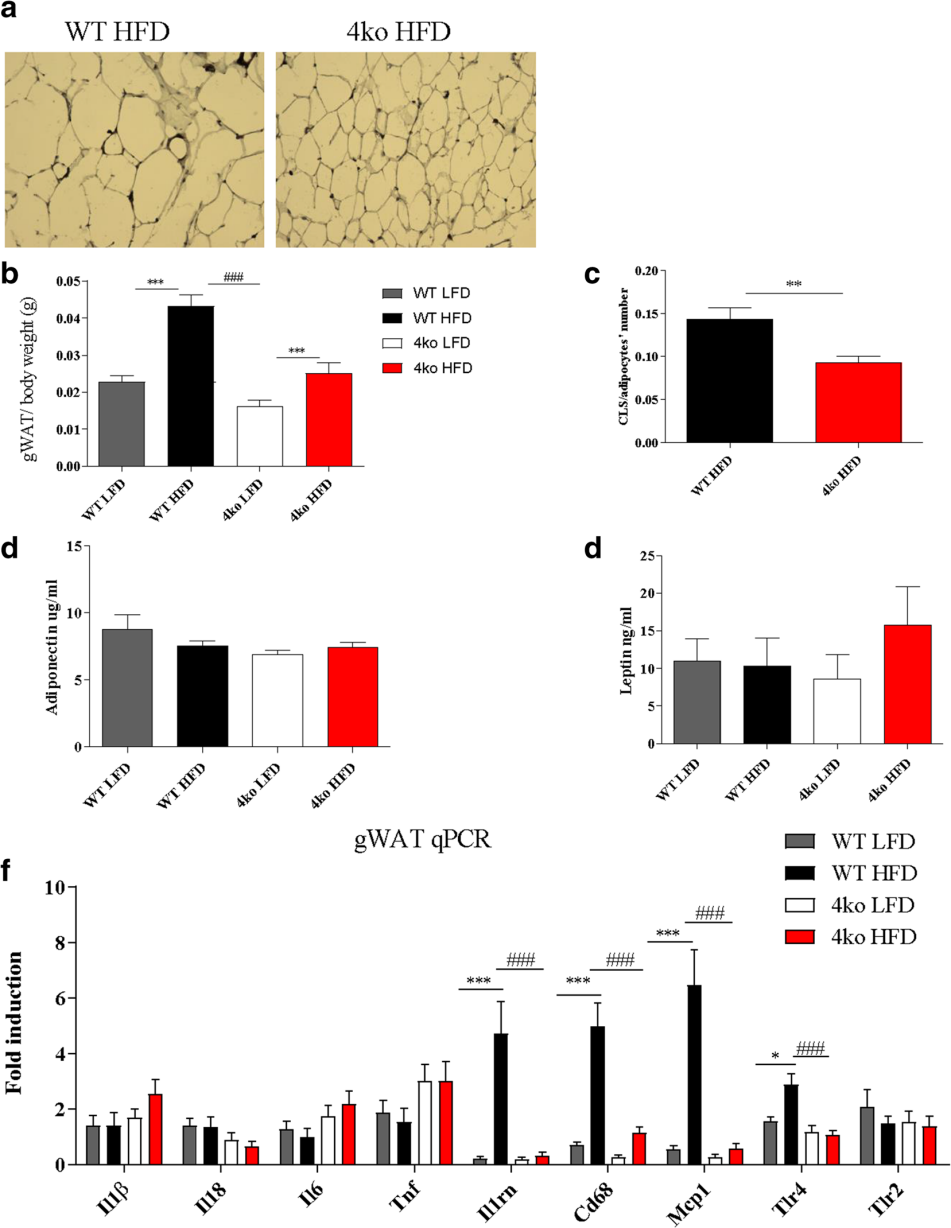
Adipose tissue inflammatory status. WT, wild-type mice; 4ko, Casp1/Casp11/NE/PR3 knockout mice. **a** Representative image of adipose tissue histology in WT mice and Casp1/Casp11/NE/PR3 knockout mice that received a HFD intervention. **b** gWAT weight reported to total body weight in the four groups of mice. **c** Crown-like structures (CLS) count in gWAT in both WT and Casp1/Casp11/NE/PR3 knockout mice that received a HFD. **d** Adiponectin concentration in plasma at the end of the diet intervention. All samples were measured in a single replicate. **e** Leptin concentration in plasma at the end of the diet intervention. All samples were measured in a single replicate. **f** mRNA fold induction of several genes in the gWAT in the four groups of mice. All samples were measured in duplicates. Data is represented as mean ± SEM. *p < 0.05, effect of the diet; **p < 0.01, effect of the diet; ***p < 0.001, effect of the diet; ###*p* < 0.001, effect of the genotype.

During obesity, several changes occur in the adipose tissue: adipocytes increase in number and size, pro-inflammatory cells infiltrate the adipose tissue leading to obesity-induced inflammation, and accumulating macrophages surround dead adipocytes forming specific complexes called crown-like structures (CLS) [26]. Interestingly, Casp1/Casp11/NE/PR3 knockout mice fed a HFD had smaller adipocytes (Fig. 4a), less adipose tissue per total body weight (0.02 ± 0.009 g *versus* 0.04 ± 0.009 g; *p* < 0.0001) (Fig. 4b), and significantly less CLS per adipocyte numbers (0.09 ± 0.02 *versus* 0.14 ± 0.04; *p* = 0.0015) than WT mice fed the same diet (Fig. 4c). These results show that Casp1/Casp11/NE/PR3 knockout mice were protected from developing obesity-induced inflammation. Next, the adiponectin and leptin plasma levels, two adipokines that are influenced by the inflammatory status, were assessed [27]. We did not observe any difference in the plasma concentration of adiponectin and leptin between the groups (Fig. 4d, e). However, plasma leptin concentration in the Casp1/Casp11/NE/PR3 knockout group that received a HFD tended to be higher when compared with WT mice on the same diet (Fig. 4e).

Expression of mRNA of inflammatory markers was also assessed in gWAT. Casp1/Casp11/NE/PR3 knockout mice that received a HFD had significantly lower mRNA levels of *Il1rn*, *Mcp1*, *Cd68*, and *Tlr4* than WT controls (Fig. 4f) and were comparable with the levels of knockout mice fed a LFD. However, no difference was observed between pro-inflammatory cytokine levels of *Il1β*, *Il18*, *Tnf*, and *Il6*, suggesting that the HFD did not induce pro-inflammatory cytokine transcription for these markers.

**Fig. 5:**
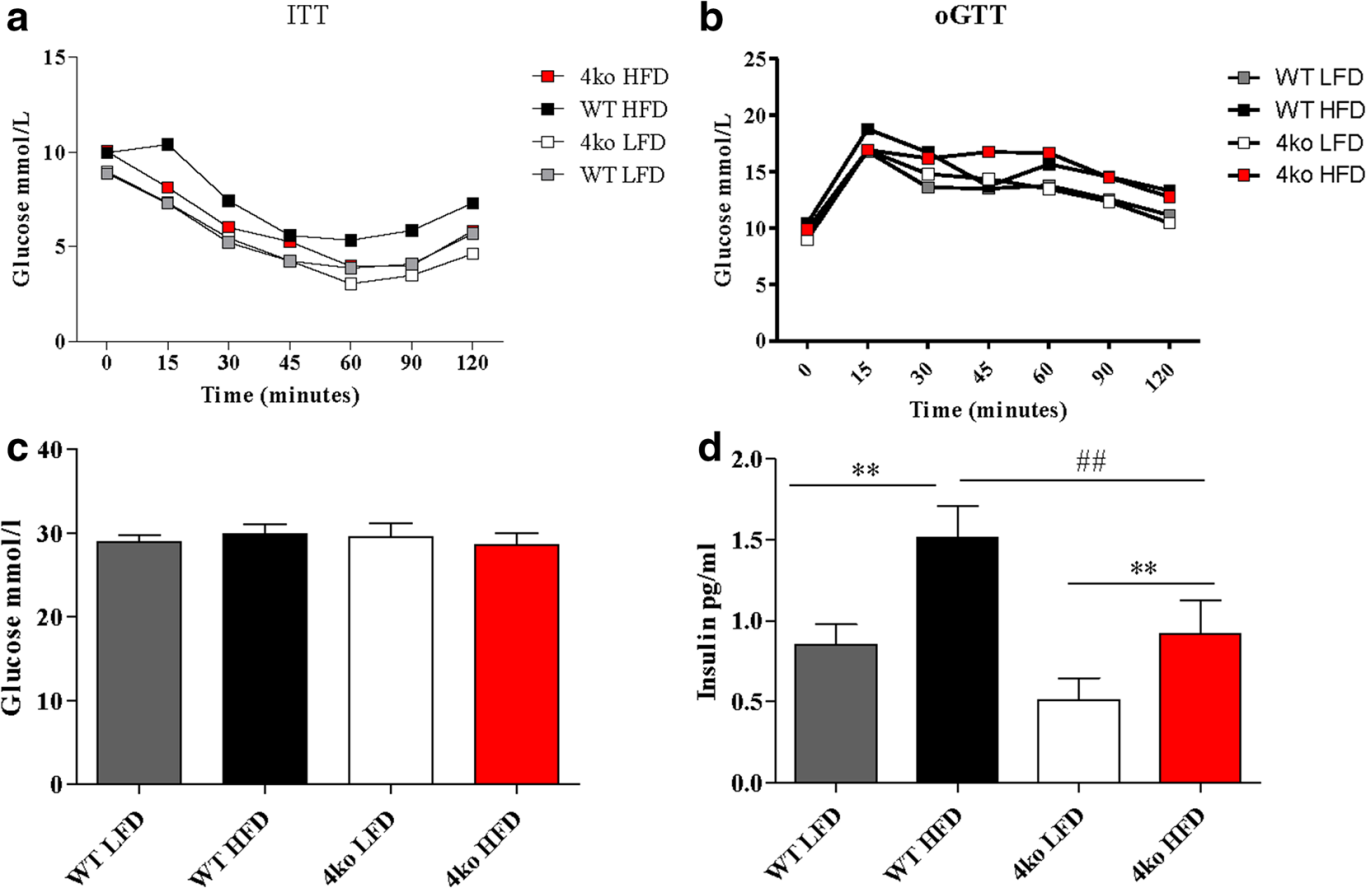
Glucose homeostasis. WT, wild-type mice; 4ko, Casp1/Casp11/NE/PR3 knockout mice. **a** Glucose blood levels during the insulin tolerance test (ITT). **b** Glucose blood levels during an oral glucose tolerance test. **c** Glucose concentration in plasma at the end of the diet intervention. **d** Insulin concentration in plasma at the end of the diet intervention. All samples were measured in a single replicate. Data is represented as mean ± SEM. **p < 0.01, effect of the diet; ##*p* < 0.01, effect of the genotype.

Another consequence of adipose tissue inflammation is the perturbation of the glycemic control and development of insulin resistance [28]. In the last 2 weeks of the diet intervention, we performed an insulin tolerance test (ITT) and an oral glucose tolerance test (oGTT) but no differences were observed between the four experimental groups (Fig. 5a, b). At the end of the diet intervention, glucose and insulin plasma concentrations were measured. No differences in glucose levels were observed between the groups (Fig. 5c). In contrast, insulin levels were significantly higher in the HFD groups when compared with the LFD groups (0.91 ± 0.73 pg/mL *versus* 0.5 ± 0.47 pg/mL for the knockout mice and 1.51 ± 0.6 pg/mL *versus* 0.85 ± 0.42 pg/mL for the WT mice; *p* < 0.01), as expected. Moreover, insulin levels in Casp1/Casp11/NE/PR3 knockout mice fed a HFD were significantly lower than insulin levels of WT mice receiving the same diet (0.91 ± 0.73 pg/mL *versus* 1.51 ± 0.6 pg/mL; *p* < 0.01), suggesting that Casp1/Casp11/NE/PR3 knockout mice might have better glycemic control (Fig. 5d).

Altogether, these data show that Casp1/Casp11/NE/PR3 knockout mice were protected for developing obesity-induced adipose tissue inflammation while no clear difference was observed for the glycemic control.

## DISCUSSION

The aim of this study was to investigate whether IL-1β activation pathways have a synergistic effect in promoting development of diet-induced NAFLD. The results show that mice lacking caspase-1 and the NSPs, PR3, and NE are protected against high-fat diet–induced weight gain, liver steatosis, and adipose tissue inflammation. Our data are in line with previous observations. Our group has previously shown that double knockout mice deficient in NE/PR3 do gain weight after HFD intervention and showed a certain degree of liver steatosis but significantly less than WT controls fed the same diet. In addition, these mice were protected against the development of adipose tissue inflammation [18]. In contrast, Dixon et al. showed that Caspase-1/11 double knockout mice developed HFD-induced obesity and liver steatosis but were protected against the development of liver inflammation and fibrosis [10, 11]. In this study, we see additional protective effects because Casp1/Casp11/NE/PR3 knockout mice do not gain weight after HFD intervention. Mice did not developed liver steatosis and despite some gain of visceral adipose tissue, they did not show signs of inflammation.

A change in the energy metabolism could also explain why quadruple knockout mice fed a HFD were protected from weight gain, liver steatosis, and gained less gWAT than WT controls. Looking at single knockout models, we know that caspase-1 knockout mice have higher fatty acid oxidation rates and their adipocytes are more metabolically active when compared with adipocytes from WT mice [12]. Also, NE knockout mice have been described to have higher energy expenditure rates, increased body temperature, and increased fatty acid oxidation [16]. In line with these results, PR3/NE knockout mice gained less weight, accumulated less triglycerides in the liver than WT mice fed a HFD, and have decreased mRNA expression of gene-encoding proteins involved in lipogenesis and fatty acid uptake, suggesting a better metabolic profile than WT mice [18]. Since our mouse model is lacking all the genes mentioned above, it is likely that these mice have also a more active basal metabolism and have an improved control of lipid metabolism than WT mice. Future studies have to be performed in order to test this hypothesis. For instance, the use of metabolic cages would help to determine how Casp1/Casp11/NE/PR3 knockout mice use energy and why they do not gain weight upon a HFD intervention.

One important consequence of obesity is the development of hypothalamic inflammation, which can further influence energy metabolism and the development of metabolic disturbances [29]; hence, it would be interesting to assess whether Casp1/Casp11/NE/PR3 knockout mice are protected from developing hypothalamus inflammation in obesity, which could also explain why these mice do not gain weight.

A limitation of our study is the fact that we did not use single caspase-1, NE, and PR3 knockout mice as controls in the diet intervention. This could have helped elucidate whether there is a cumulative protective effect against obesity-induced metabolic disturbance. Another limitation is the fact that the diet we used lead to the development of liver steatosis, an early stage of NAFLD. Looking at the gene expression profiles in both liver and adipose tissue, we do not see any difference in levels of pro-inflammatory cytokines *Il1β*, *Il18*, *Tnf*, and *Il6*, but we do see some upregulation of the chemokine *Mcp1* in both quadruple knockout mice and WT that received a HFD. Additionally, we see an upregulation of anti-inflammatory cytokine gene *Il1rn* and surface marker genes *Cd68* and *Tlr4* in the WT mice that received HFD. All these results suggest that there is an induction of inflammation during the HFD intervention but not strong enough to stimulate pro-inflammatory cytokine production. A NASH-inducing diet leads to the development of a more severe form of inflammation and we should therefore be able to observe a difference for *Il1β* and *Il18* mRNA levels between Casp1/Casp11/NE/PR3 knockout mice and WT mice.

An interesting experiment would be to investigate whether blocking pharmacologically multiple IL-1β activation pathways during a HFD intervention is also able to prevent liver damage. So far, we can conclude that mice deficient for IL-1β activation pathways are protected against NAFLD and liver steatosis.

## CONCLUSION

In this study, we developed a unique mouse model, which can be used to study both metabolic and inflammatory diseases. Additionally, we observed that blocking IL-1β activation pathways protects against HFD-induced obesity and associated liver steatosis in mice. Further mechanisms and therapeutic potential in patients with NAFLD need to be explored.

## Electronic Supplementary Material

ESM 1 (DOCX 12 kb)
