# Supplementary material for: Mice Deficient in the IL-1β Activation Genes Prtn3, Elane, and Casp1 Are Protected Against the Development of Obesity-Induced NAFLD
Source: Inflammation. 2020 Jan 30;43(3):1054–64. doi: 10.1007/s10753-020-01190-4 (PMC7280336; doi:10.1007/s10753-020-01190-4)
Supplement: Supplementary file 1 — (DOCX 12 kb) [file 10753_2020_1190_MOESM1_ESM.docx]

| **Gene name** | **Primer sequence forward** | **Primer sequence reverse** |
| --- | --- | --- |
| ***36b4*** | AGCGCGTCCTGGCATTGTGTGG | GGGCAGCAGTGGTGGCAGCAGC |
| ***Il1β*** | GCCGTCTTTCATTACACAGGA | ACAAGGAGAACCAAGCAACG |
| ***Il18*** | TGTCGCAGGAATAAAGATGGCT | CCTTGGTCAATGAAGAGAACTTGGT |
| ***Il33*** | TATCCACGGGATTCTAGGAAGAG | TGGTCATTGTATGTACTCAGGGA |
| ***Il6*** | ACAAGGAGAACCAAGCAACG | CCGGAGAGGAGACTTCACAG |
| ***Tnf*** | GTGGGTGAGGAGCACGTAGT | ACGGCATGGATCTCAAAGAC |
| ***Il1rn*** | AAATCTGCTGGGGACCCTAC | TGAGCTGGTTGTTTCTCAGG |
| ***Cd68*** | CCAATTCAGGGTGGAAGAAA | CTCGGGCTCTGATGTAGGTC |
| ***Mcp1*** | CCCAATGAGTAGGCTGGAGA | TCTGGACCCATTCCTTCTTG |
| ***Tlr2*** | AACCTCAGACAAAGCGTCAAATC | ACCAAGATCCAGAAGAGCCAAA |
| ***Tlr4*** | TTCCTTCTTCAACCAAGAACATAGATC | TTGTTTCAATTTCACACCTGGATAA |

**Supplementary Table 1. List of primers used for qPCR**
